# A low-cost approach to upskilling tutors in frontline health care worker production value chain

**DOI:** 10.4102/phcfm.v16i1.4597

**Published:** 2024-08-12

**Authors:** Anddy Omoluabi, Linda Ugalahi, Olufolake Akeju, Emmanuel Aiyenigba

**Affiliations:** 1 Health Workforce Management Activity (USAID-Funded), Banyan Global, Abuja, Nigeria

**Keywords:** TutorConnect, nurse tutors, PHC tutors, preceptors, primary healthcare

## Abstract

Nigeria, like many countries, struggles with a shortage of healthcare professionals including frontline healthcare providers at the primary healthcare (PHC) level. While the country is pitched towards producing more healthcare professionals, the existing infrastructure to produce them is inadequate. Producing healthcare professional with the required skills to deliver quality services is impeded by several factors including the use of outdated curricula for their training, low application of technology in teaching, and weak tutor capacity that is worsened by the paucity and high cost of opportunities for tutor continuing professional development. To address these issues, the Health Workforce Management Activity (HWM), an initiative of the United States Agency for International Development (USAID), in 2023 designed and implemented a low-cost programme called TutorConnect that offered low-cost continuing professional development to tutors. TutorConnect is a Zoom-based monthly training programme that is facilitated by subject matter experts. The programme offered topics spanning effective learning, enhanced instructional design, and improved student engagement in the 14 months of its operation with over 700 tutors from more than 10 states in Nigeria that benefited from it. Utilising WhatsApp for additional support and peer-to-peer learning was crucial to providing more hands-on support, and institution-specific solutions. This low-cost approach to build competencies enabled access to continuing professional development by tutors, limiting effects of location and finances as barriers to continuing professional development. Developing the teaching capacity of tutors is pivotal to enhancing the quality of frontline healthcare worker training.

## Introduction

Primary healthcare (PHC) is recognised globally as the bedrock of effective health systems.^1^ Adequate training of frontline health professionals is pivotal to improving the quality and accessibility of care.^2^ Like several other countries, Nigeria is grappling with a critical shortage of healthcare workers and providers.^3^ It has therefore adopted several strategies in line with global trends and local realities to address the hindrances to achieving universal health coverage. While the country has implemented several initiatives to bolster the production of frontline PHC workforce with varying degrees of success, not much attention has been given to the downstream factors that facilitate the production of midwives, nurses, and community health professionals, in particular, so that they are fit for purpose to tackle the increasing complexities of the country’s health issues in the face of a rapidly growing population and investment deficits in health infrastructure.^4^

Producing these professionals with the required quality and competencies is impeded by several challenges including the use of dated training curricula, low utilisation of technology in teaching methodologies, and inadequate opportunities for tutor development programmes, among others.^5^

## The Health Workforce Management Activity initiative

The United States Agency for International Development, Nigeria Health Workforce Management Activity (HWM) has implemented an innovative and cost-saving strategy for strengthening the capacity of nurse educators, PHC tutors of pre-service health training institutions, and clinical preceptors of pre-service health workers. Called TutorConnect, the programme is facilitated by subject matter experts who offer topics spanning effective learning, enhanced instructional design, and improved student engagement. The TutorConnect sessions were held monthly and characterised by the administration of pre- and post-tests to demonstrate knowledge improvement. This is in addition to the use of participative methods that facilitate reflections and elicit practical experiences. A typical TutorConnect session commences with a pre-session assessment of tutors, followed by an interactive session on a specific topic. These topics were designed based on outcomes of a situation analysis conducted by HWM to identify gaps in competencies of the tutors. Polls, breakout rooms and games were approaches utilised to enhance participation. At the end of each session, post-session assessment and feedback were conducted.

To foster continuous engagement and build a sense of community, the intervention utilised WhatsApp groups as a supportive mechanism that fostered peer-to-peer learning and experience sharing. Over 700 tutors from more than 10 states in Nigeria participated in the sessions. Compared to the associated costs for travelling to attend similar training programmes, which could be between 250 000 naira and 300 000 naira per tutor for a 5-day training programme, tutors who attended TutorConnect only bore the costs for internet connection of around 10 000 naira per tutor for five sessions. After 14 months of implementing TutorConnect using Zoom, HWM realised that several of the tutors struggled to join and maintain a steady internet connection during the session. This is in addition to feedback received from some tutors about the difficulty with the time of the day for the sessions. In response, HWM grew the TutorConnect programme to an online self-paced learning system in partnership with a local Nigerian private organisation that offers e-learning solutions. To reinforce learning and create a community of practice, HWM created and facilitated conversations among the tutors on a monthly Microsoft Teams-based community call session.

## What has changed?

Health Workforce Management Activity interviewed some tutors who participated in TutorConnect sessions and students in institutions where these tutors teach and observed several short-term changes attributed to both versions of TutorConnect. These include a self-reported improvement in the attitude of the tutors, increased knowledge about the new technologies for instructional design, effective teaching strategies, curriculum implementation, ethics and professional standards, and reflective practice. On the part of the leadership of the institutions, several of them have reported to HWM through verbal feedback about increased awareness of the requirements for establishing an enabling learning environment and an enhanced level of engagement between the tutors and the senior management. These changes have led to more effective coordination of learning delivery activities in some of the schools.

From the perspective of the students, the transformation in the knowledge and attitude of the tutors has translated into a significant improvement in the skills demonstrated by the tutors and preceptors as they utilise improved teaching methods and approaches. Some of the areas where students have noticed the most significant improvement include the level of engagement, more proactiveness in classroom management, and increased use of technology such as use of PowerPoint presentation and school-based learning management systems in their delivery.

## Conclusion

While Nigeria emphasises the ramping up of the production of frontline healthcare workers, the quality of the training that they receive is now more than ever important. Prioritising tutor development is crucial for enhancing the quality of frontline healthcare worker training. The innovative approaches developed by HWM shows potential for low-cost and scalable methods for the training of tutors.
